# Combination of genetic engineering and random mutagenesis for improving production of raw-starch-degrading enzymes in *Penicillium oxalicum*

**DOI:** 10.1186/s12934-022-01997-w

**Published:** 2022-12-24

**Authors:** Shuai Zhao, Ming-Zhu Tan, Rui-Xian Wang, Fa-Ting Ye, Yuan-Peng Chen, Xue-Mei Luo, Jia-Xun Feng

**Affiliations:** State Key Laboratory for Conservation and Utilization of Subtropical Agro-Bioresources, Guangxi Research Centre for Microbial and Enzyme Engineering Technology, College of Life Science and Technology, Guangxi University, Nanning, China

**Keywords:** Raw starch-degrading enzymes, Genetic engineering, Random mutagenesis, *Penicillium oxalicum*, Biorefining

## Abstract

**Background:**

Raw starch-degrading enzyme (RSDE) is applied in biorefining of starch to produce biofuels efficiently and economically. At present, RSDE is obtained via secretion by filamentous fungi such as *Penicillium oxalicum*. However, high production cost is a barrier to large-scale industrial application. Genetic engineering is a potentially efficient approach for improving production of RSDE. In this study, we combined genetic engineering and random mutagenesis of *P. oxalicum* to enhance RSDE production.

**Results:**

A total of 3619 mutated *P. oxalicum* colonies were isolated after six rounds of ethyl methanesulfonate and Co^60^-γ-ray mutagenesis with the strain A2-13 as the parent strain. Mutant TE4-10 achieved the highest RSDE production of 218.6 ± 3.8 U/mL with raw cassava flour as substrate, a 23.2% compared with A2-13. Simultaneous deletion of transcription repressor gene *PoxCxrC* and overexpression of activator gene *PoxAmyR* in TE4-10 resulted in engineered strain GXUR001 with an RSDE yield of 252.6 U/mL, an increase of 15.6% relative to TE4-10. Comparative transcriptomics and real-time quantitative reverse transcription PCR revealed that transcriptional levels of major amylase genes, including raw starch-degrading glucoamylase gene *PoxGA15A*, were markedly increased in GXUR001. The hydrolysis efficiency of raw flour from cassava and corn by crude RSDE of GXUR001 reached 93.0% and 100%, respectively, after 120 h and 84 h with loading of 150 g/L of corresponding substrate.

**Conclusions:**

Combining genetic engineering and random mutagenesis efficiently enhanced production of RSDE by *P. oxalicum*. The RSDE-hyperproducing mutant GXUR001 was generated, and its crude RSDE could efficiently degrade raw starch. This strain has great potential for enzyme preparation and further genetic engineering.

**Supplementary Information:**

The online version contains supplementary material available at 10.1186/s12934-022-01997-w.

## Background

Bioethanol produced by starch biorefining, specially corn starch, is a major source of renewable biofuels in many countries [1]. Typically, starch biorefining for bioethanol production comprises three steps: (1) gelatinisation of raw starch flour and liquefaction with α-amylase at high temperature (80–105 °C); (2) saccharification with glucoamylase at 60–65 °C to generate sugar syrup; (3) fermentation by *Saccharomyces cerevisiae* to ethanol. Of these, gelatinisation and liquefaction require high energy input, accounting for 10–20% of the bioethanol price, which negatively affects the competitiveness of bioethanol against fossil fuels [2], and hence our ability to achieve carbon neutrality.

Interestingly, the uncooked raw starch can be directly digested into glucose that is fermented into bioethanol, by raw starch-degrading enzyme (RSDE) below the temperature required for gelatinisation through synergistic cooperation, specifically raw starch-degrading glucoamylase (RSDG) and raw starch-degrading α-amylase. The raw starch-degrading α-amylase randomly breaks α-1,4-glycosidic linkages inside starch granules to expose non-reducing ends for RSDG, while RSDG cleaves both α-1,4- and α-1,6-glycosidic bonds to subsequently release glucose. The high cost of RSDG is considered a limiting factor for bioethanol production from raw starch biorefining [3, 4].

Although a number of amylases have been identified and characterised, only a few RSDEs are known to contain starch-binding domains [5]. In general, RSDEs are mainly biosynthesised by filamentous fungi, such as members of the genera *Penicillium* and *Aspergillus*, but yields are quite low [2]. Several approaches, including physical and/or chemical mutagenesis, optimisation of cultivation parameters, and genetic modification have been employed to improve enzymatic yields [6–8]. However, a single approach often has limited success.

Previous work identified a potential RSDG, PoxGA15A, in *Penicillium oxalicum* strain GXU20 [9], which exhibited broad substrate specificity and high pH stability. Application of PoxGA15A in simultaneous saccharification and fermentation, alongside a commercial α-amylase, led to high fermentation efficiency (> 90%) with raw flour from either corn or cassava as feedstock [10]. Furthermore, engineering PoxGA15A incorporating a strong promoter and signal peptide, as well as mutagenesis by atmospheric and room temperature plasma (ARTP) and ethyl methanesulfonate (EMS), were used to enhance crude RSDE production by *P. oxalicum*, [7, 8]. However, the RSDE production has not met the requirement for raw starch biorefinery.

Biosynthesis of amylase is precisely regulated by transcription factors (TFs). Several TFs regulate the expression of amylase genes, including transcriptional activator AmyR and repressor CreA in *P. oxalicum* [4]. Unfortunately, only few TFs are known to regulate RSDE gene expression. For example, CxrC, NsdD and HmbB negatively regulate expression of the *PoxGA15A* gene in *P. oxalicum* [11–13], while POX01907 positively regulated *PoxGA15A* expression [14]. However, the effects of TF cooperation on RSDE production have not been reported.

Here, we employed mutagenesis by Co^60^ and EMS combined with TF-based genetic engineering to improve production of RSDE, using *P. oxalicum* mutant A2-13 as the parent strain, derived from mutant OX*PoxGA15A* in which *PoxGA15A* is overexpressed compared with wild-type strain HP7-1 [7, 8]. We then evaluated the digestion efficiency of raw flours from both corn and cassava using crude RSDE from the resulting mutant.

## Results and discussion

### Combined Co^60^ and EMS mutagenesis and isolation of RSDE-hyperproducer TE4-10

In previous work, *P. oxalicum* mutant A2-13, a strain producing high levels of RSDE, was generated through multiple rounds of random mutagenesis with ARTP and/or EMS from parent strain OX*PoxGA15A* using a two-layer agar gel diffusion method [7]. In the OX*PoxGA15A*, the RSDG gene *PoxGA15A*, controlled via the inducible promoter P_PoxEgCel5B_ by cellulose, was overexpressed in parental strain ∆*PoxKu70* [8]. Here, we employed both random mutagenesis and genetic engineering to improve the yield of RSDE using A2-13 as the parent strain. Random mutagenesis included four rounds of EMS mutagenesis and two rounds of Co^60^-γ-ray mutagenesis. Prior to random mutagenesis, conidia of mutant A2-13 were treated with a final concentration of 1.2% EMS for 10 h, resulting in a 96.05% lethality rate. Co^60^-γ-ray irradiation mutagenesis was carried out at five doses from 0.6 KGy to 3.0 KGy. At 0.6 KGy, the lethality rate of mutant TE2-23 reached 92.74%. By contrast, the lethality rate of mutant Co1-17 was 87.65% at 1.6 KGy (Additional file 1: Fig. S1).

**Fig. 1:**
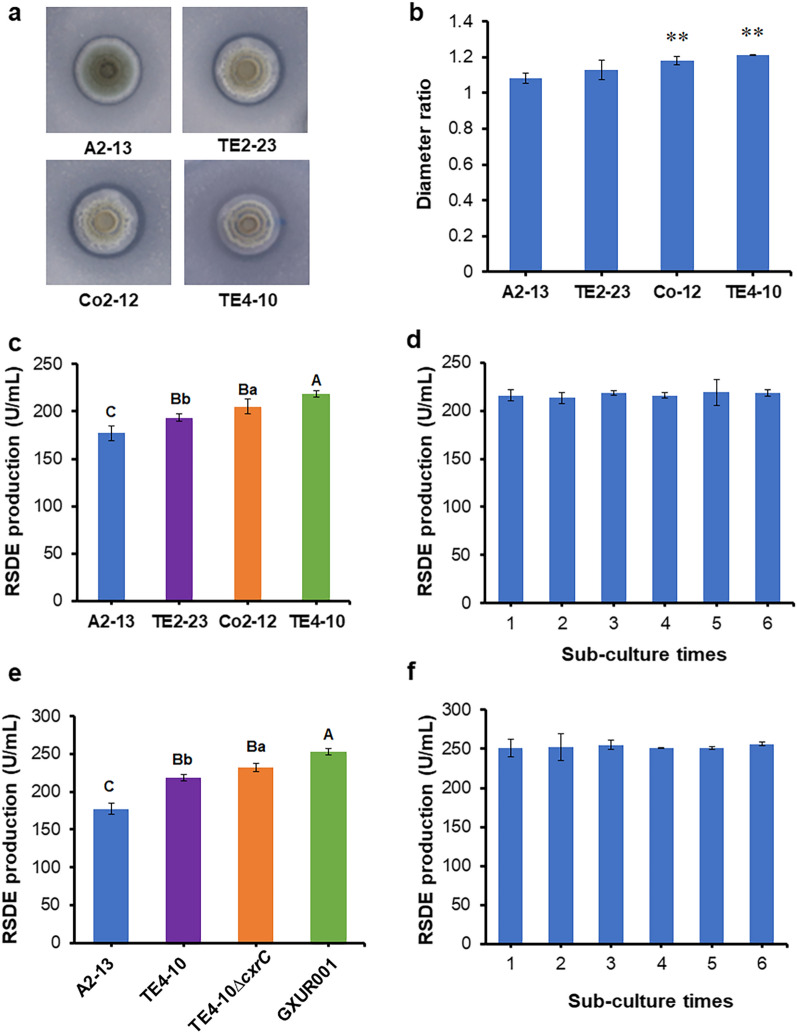
Analysis of *Penicillium oxalicum* mutants generated by random mutagenesis and genetic engineering. (**a**) Hydrolysis zones of fungal colonies. (**b**) Diameter ratio of clear zones vs. colonies: **p < 0.01 indicates significant differences between mutants and parent strain A2-13. (**c**) Raw starch-degrading enzyme (RSDE) production of mutants obtained by random mutagenesis after culture for 8 days on wheat bran plus Avicel. (**d**) RSDE production of mutant TE4-10 sequentially sub-cultured six times for 8 days each time on wheat bran plus Avicel. (**e**) RSDE production of engineered strains cultured for 8 days on wheat bran plus Avicel. Strains A2-13 and TE4-10 served as controls. In panels c and e, capital and small letters indicate *p* < 0.01 and *p* < 0.05, respectively. Different letters reveal significant differences between mutant and parental strains, evaluated by one-way ANOVA. (f) RSDE production of engineered strain GXUR001 sequentially sub-cultured six times for 8 days each time on wheat bran plus Avicel. Results are mean ± standard deviation. All tests were performed in triplicate

After several rounds of physical–chemical mutagenesis, 3619 colonies were obtained, of which 108 displaying a large ratio between colonies and clear zones were selected for further evaluation. Eventually, we identified three mutants (TE2-23, Co2-12 and TE4-10) producing more RSDE than A2-13. The ratio of the diameter between colonies and clear zones for mutant TE4-10 was the largest (Fig. 1a, b). The RSDE yields of mutants TE2-23, Co2-12 and TE4-10 on day 8 of culture in medium containing wheat bran plus Avicel were 193.29 ± 3.80, 205.36 ± 7.61 and 218.55 ± 3.83 U/mL, respectively, using raw cassava flour as substrate, an increase by 8.94%, 15.75% and 23.18%, respectively, compared with A2-13 (Fig. 1c). Avicel added in the medium can induce the promoter P_PoxEgCel5B_ controlling the engineered RSDG gene *PoxGA15A*. Furthermore, the stability of mutant TE4-10 was assessed based on RSDE production. The results revealed that production of RSDE exhibited no significant alteration after six rounds of sub-culture (Fig. 1d).

### Genetic engineering of transcriptional regulators to improve RSDE production

Expression of enzyme genes can be enhanced significantly using strong promoters, and this depends on regulation by TFs. Genetic engineering based on regulatory networks formed by numbers of TFs is an efficient method for enhancing enzyme production. PoxCxrC and PoxAmyR respectively repress and stimulate the transcription of key amylase genes, *PoxGA15A* and *PoxAmy13A* [4, 13]. Therefore, it will be interesting to explore the effects of combining these TFs for RSDE production in *P. oxalicum*.

We engineered *P. oxalicum* strain TE4-10 to obtain TE4-10Δ*cxrC*::*amyR*, renamed GXUR001, in which *PoxCxrC* was deleted and *PoxAmyR* was simultaneously overexpressed, and verified the strain by PCR analysis (Additional file 2: Fig. S2). When cultivated in medium containing wheat bran plus Avicel as carbon sources for 8 days, RSDE production of GXUR001 reached 252.58 ± 4.24 U/mL with raw cassava flour as hydrolysis substrate, 42.36% and 15.55% higher than mutants A2-13 and TE4-10, respectively (Fig. 1e). Moreover, the genetic stability of GXUR001 was evaluated based on RSDE production. The results revealed that RSDE production of GXUR001 showed no significant alteration after six successive sub-culture steps (Fig. 1f). Compared with results of previous studies (Table 1), RSDE production by GXUR001 using culture of shake flask was the highest yet reported, with raw cassava flour as hydrolysis substrate. Notably, enzymatic activity specifically depended on the hydrolysis substrate. Therefore, RSDE yields reported for other raw starch substrates are not directly comparable.

**Table 1:** Comparison of raw-starch-degrading enzyme production between *Penicillium oxalicum* engineered strain GXUR001 and other fungal strains

Microorganisms	Starch substrate	Enzyme production (U/mL)	References
*P. oxalicum* GXUR001	Nature raw cassava flour	252.58	This study
*P. oxalicum* A2-13	Nature raw cassava flour	190.96	[7]
*P. oxalicum OXPoxGA15A*	Nature raw cassava flour	100.97	[7]
*P. oxalicum OXPoxGA15A*	Processed raw cassava starch	241.6	[8]
*P. oxalicum* Δ*PoxKu70*	Raw cassava starch	55.1	[8]
*P. oxalicum* GXU20	Raw cassava starch	20	[9]
*Geobacillus* sp. 4 J	Raw corn starch	39.6	[15]
	Raw soluble starch	28.6	
*Aspergillus flavus NSH9*	Raw sago starch	10.44	[16]
*Aspergillus fumigatus*	Raw corn starch	25	[17]
*Aspergillus sp.* MZA-3	Raw cassava starch	3.3	[18]

However, unexpectedly, PoxCxrC was found to more weakly inhibit RSDE production in TE4-10 compared with ∆*PoxKu70*. The ∆*PoxCxrC* mutant exhibited 1.5- to 1.8-fold enhanced production of RSDE relative to ∆*PoxKu70* when cultured on soluble corn starch for 2 to 4 days, respectively [13]. Mutant TE4-10 was derived from ∆*PoxKu70* through multiple rounds of physical–chemical random mutagenesis and overexpressing the *PoxGA15A* gene, which might alter the regulatory network controlling the biosynthesis of amylases [7, 8]. Additionally, the distinct effects caused as a result of deletion of *PoxCxrC* in the ∆*PoxKu70* and TE4-10 on RSDE production might be resulted from induction by different carbon sources.

### Properties of crude RSDE secreted by engineered strain GXUR001

**Fig. 2:**
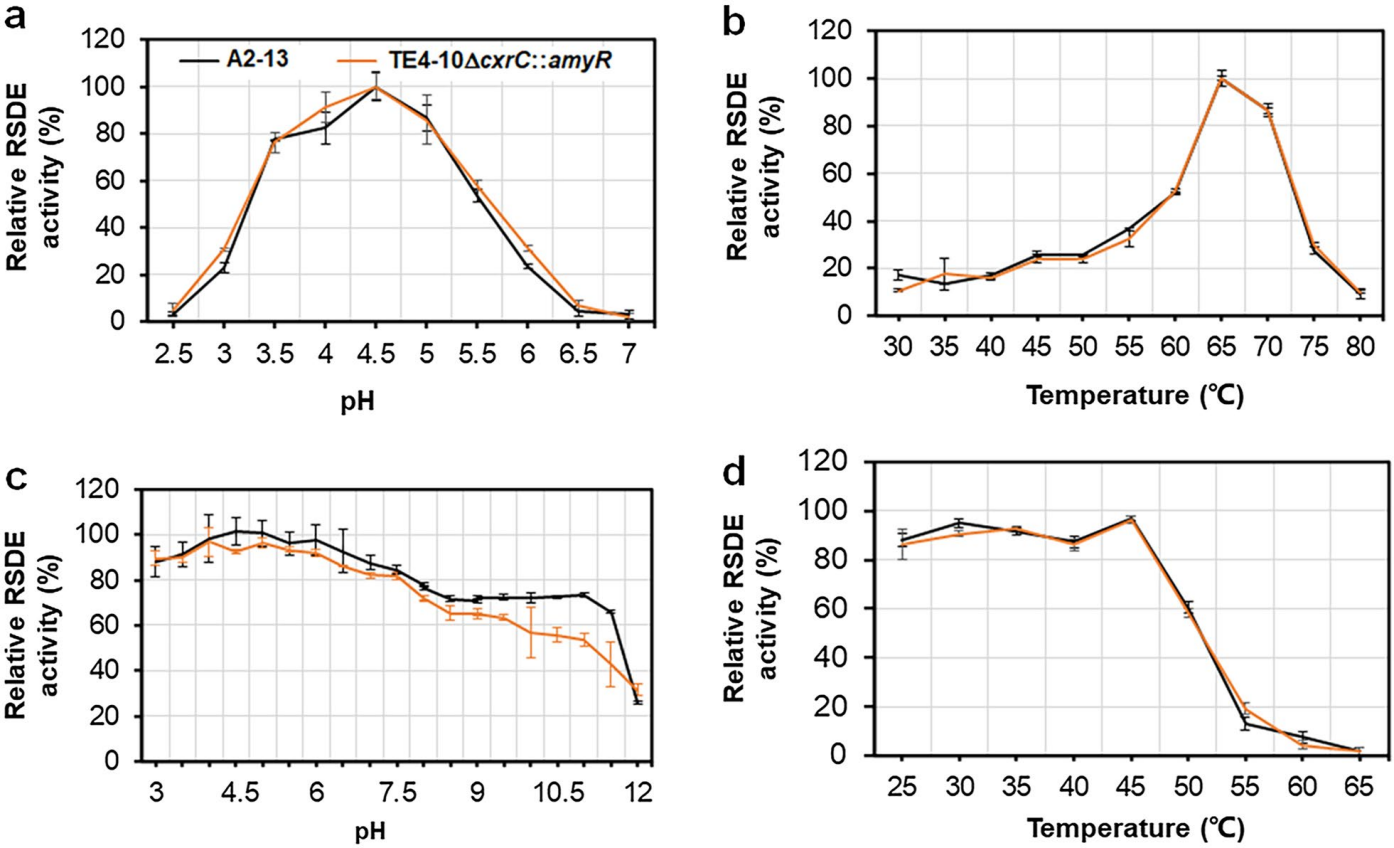
Effects of pH and temperature on the activity of RSDE from *Penicillium oxalicum* mutant GXUR001 and parent strain A2-13. (**a**) pH profile. (**b**) Temperature profile. (**c**) pH stability. (**d**) Thermal stability. Crude RSDE is prepared from *P. oxalicum* strains cultivated on wheat bran plus Avicel for 8 days under optimal culture conditions. In panels a and b, the highest RSDE activity of GXUR001 and A2-13 was set as 100%. In panels **c** and **d**, the RSDE activity of untreated GXUR001 and A2-13 was set as 100%. Results are mean ± standard deviation. Each experiment included three biological replicates

In order to examine the effects of EMS and Co^60^-γ mutagenesis and genetic engineering on the features of crude RSDE, the optimal pH and thermostability were determined. The optimal pH was 4.5 and the optimal temperature was 65 °C (Fig. 2a, b). The pH and thermostability of GXUR001 RSDE was in accordance with those of A2-13, apart from a stronger tolerance of alkaline conditions (Fig. 2c, d).

### Alteration of gene expression in GXUR001

The abundance of mRNAs plays a critical role in boosting cellulase and xylanase content in fungi. Here, RNA sequencing (RNA-seq) and RT-qPCR were employed to probe alteration of gene expression in genetically engineered GXUR001 cultured on wheat bran plus Avicel, especially amylase-encoding genes, with mutant TE4-10 serving as a control. After pre-growing in glucose medium for 24 h, mycelia were collected and placed on induction medium containing wheat bran plus Avicel for 24 h. RNA-seq data yielded 22 million clean reads for each sample, and each read was ~ 50 bp in length. Over 98% of clean reads could be matched against the genome of *P. oxalicum* wild-type strain HP7-1 [19]. Quality evaluation of RNA data revealed a very high Pearson correlation coefficient (*r* > 0.96) among three biological replicates for each strain (Additional file 3: Fig. S3), indicating that the transcriptome data were credible.

**Fig. 3:**
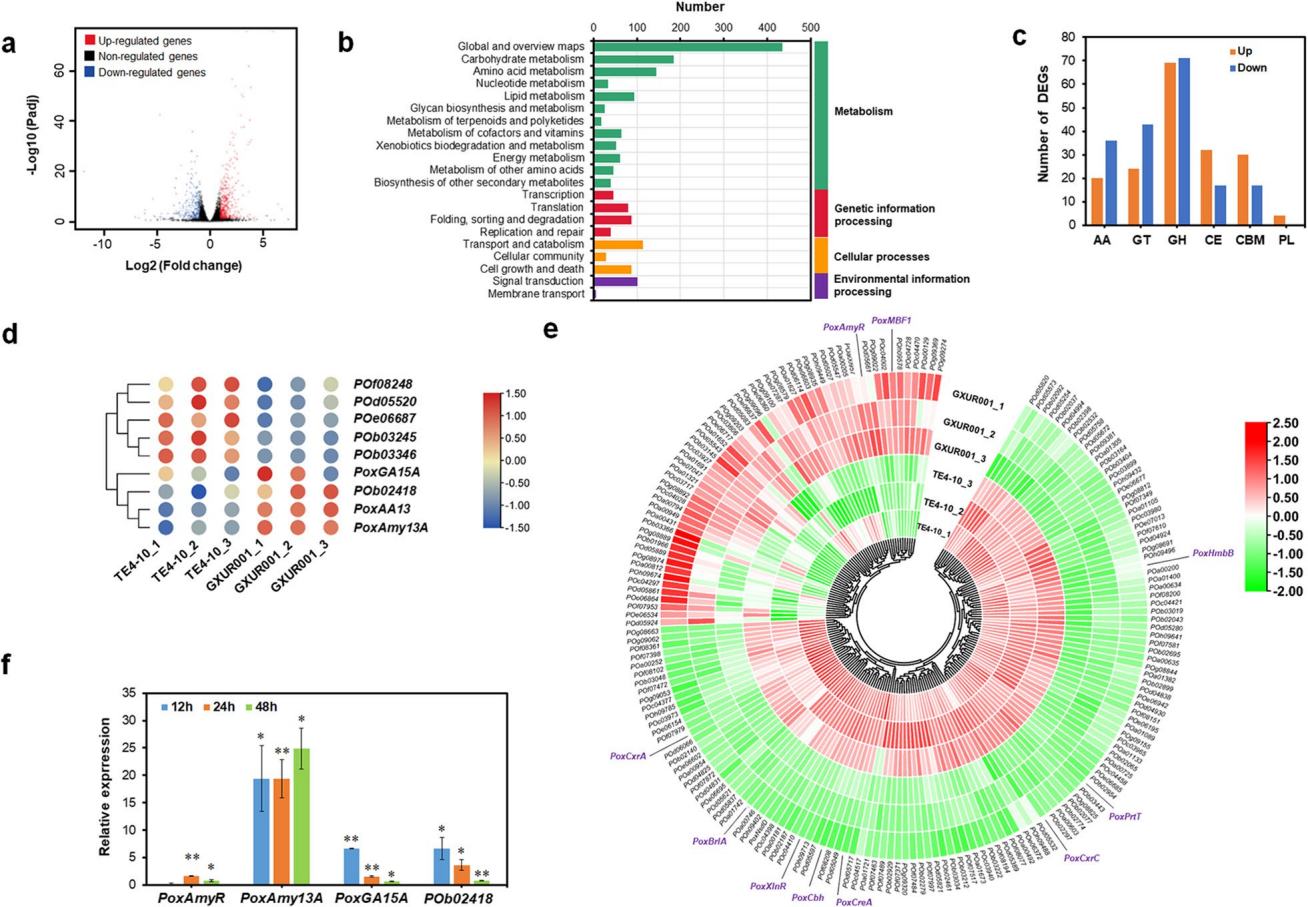
Analysis of gene expression in engineered *Penicillium oxalicum* strain GXUR001 relative to parental strain TE4-10. (**a**) Volcano plot indicating differentially expressed genes (DEGs). (**b**) Analysis of DEG-encoding protein functions based on Kyoto Encyclopedia of Genes and Genomes annotation. (**c**) DEGs encoding carbohydrate-active enzymes. AA, auxiliary activities; GT, glycosyltransferase; GH, glycoside hydrolase; CE, carbohydrate esterase; CBM, carbohydrate-binding module; PL, polysaccharide lyase. (**d**) DEGs encoding major amylases and lytic polysaccharide monooxygenase. Raw starch-degrading glucoamylase PoxGA15A; Glucoamylase POb02418; α-amylases PoxAmy13A and POb03245; α-glucosidases POf08248 and POe06687; 1,4-α-glucan-branching enzyme POd05520; Lytic polysaccharide monooxygenase PoxAA13. (**e**) DEGs encoding transcription factors. (**f**) Real-time quantitative reverse transcription PCR assays indicating relative expression of key amylase genes and regulatory gene *PoxAmyR*. Results are mean ± standard deviation: **p < 0.01 and **p < 0.05 indicate significant differences between GXUR001 and TE4-10, analysed by Student’s *t*-test. Each experiment included three biological replicates

With a threshold of *p* < 0.05, 3292 differentially expressed genes (DEGs) were found in GXUR001 relative to the parental strain TE4-10 (Fig. 3a; Additional file 4: Table S1), of which 1524 were upregulated (0.2 ≤ Log2 fold change ≤ 6.8) and 1768 downregulated (− 0.2 ≤ Log2 fold change ≤  − 11.9). Analysis of metabolic pathways using the Kyoto Encyclopedia of Genes and Genomes database showed that DEG-encoded proteins were mainly related to metabolism (Fig. 3b).

Among the 3292 DEGs, 324 were found to encode carbohydrate-active enzymes, belonging to various families (Fig. 3c), including auxiliary oxidoreductase (56), glycosyltransferase (67), glycoside hydrolase (140), carbohydrate esterase (49), carbohydrate-binding module (44) and polysaccharide lyase (4). Comparative analysis revealed 152 DEGs with increased expression in GXUR001 compared with TE4-10. These included three key amylase genes, namely RSDG gene *PoxGA15A*, glucoamylase gene *POX_b02418* and α-amylase gene *PoxAmy13A*, transcription levels of which were increased 27.1–207.5% in GXUR001 (Fig. 3d). Additionally, *POX_g09282* (*PoxAA13*) encoding lytic polysaccharide monooxygenase exhibited 2.8-fold enhanced expression in GXUR001. AA13 catalyses oxidative cleavage of insoluble starch. Deletion of *AA13* in *Aspergillus nidulans* seriously impaired the degradation of resistant starch, but showed no effects against soluble starch [20]. Unexpectedly, the transcriptional abundance of two α-glucosidase genes (*POX_f08248* and *POX_e06687*), 1,4-α-glucan-branching enzyme gene *POX_d05520*, and α-amylase gene *POX_b03245* were downregulated in GXUR001 (Fig. 3d).

Moreover, 187 DEGs encoding putative TFs were identified, approximately two-thirds of which were downregulated in GXUR001 relative to TE4-10. As expected, expression of *PoxAmyR* exhibited a 62.75% increase, whereas *PoxCxrC* expression wasn’t detected. The transcriptional repressor gene *PoxCreA* showed 47.6% reduced expression. C2H2 protein CreA that mediates carbon catabolite repression impaired the transcription of amylase genes, either indirectly by repressing the expression of regulatory genes including *amyR*, or directly by binding the promoters of amylase genes [4]. Surprisingly, some known transcriptional activator genes involved in amylase biosynthesis, including *PoxPrtT* [21], *PoxHmbB* [12] and *PoxNsdD* [11], displayed decreased expression by 22.6–32.2% in GXUR001 (Fig. 3e). Therefore, expression of major amylase genes in GXUR001 was altered via the coordination of many regulatory genes when cells were cultured on wheat bran plus Avicel.

To further confirm the RNA-seq results, expression levels of four important genes, *PoxGA15A*, *PoxAmy13A*, *POX_b02418* and *PoxAmyR*, were examined by RT-qPCR. *P. oxalicum* strains GXUR001 and TE4-10 were induced on wheat bran plus Avicel for 12–48 h after transfer from glucose, and the resulting mycelia were subjected to RNA extraction. The results revealed that the transcriptional abundances of all tested genes in GXUR001 were enhanced throughout the induction period, by 61.7–2492.0%, compared with those in TE4-10, except *PoxAmyR* at 12 h which showed no significant alteration (Fig. 3f).

Interestingly, although a remarkable increase in expression of major amylase genes was achieved, RSDE production by GXUR001 remained unsatisfactory. Previous studies revealed that high mRNA levels were a prerequisite for enhanced amounts of secreted proteins, but a strong translation machinery [22] and transport system were also essential. Therefore, in future work, transcription, translation and transportation should be simultaneously investigated to improve RSDE production by filamentous fungi.

### Phenotypic analyses of *P. oxalicum* mutants

**Fig. 4:**
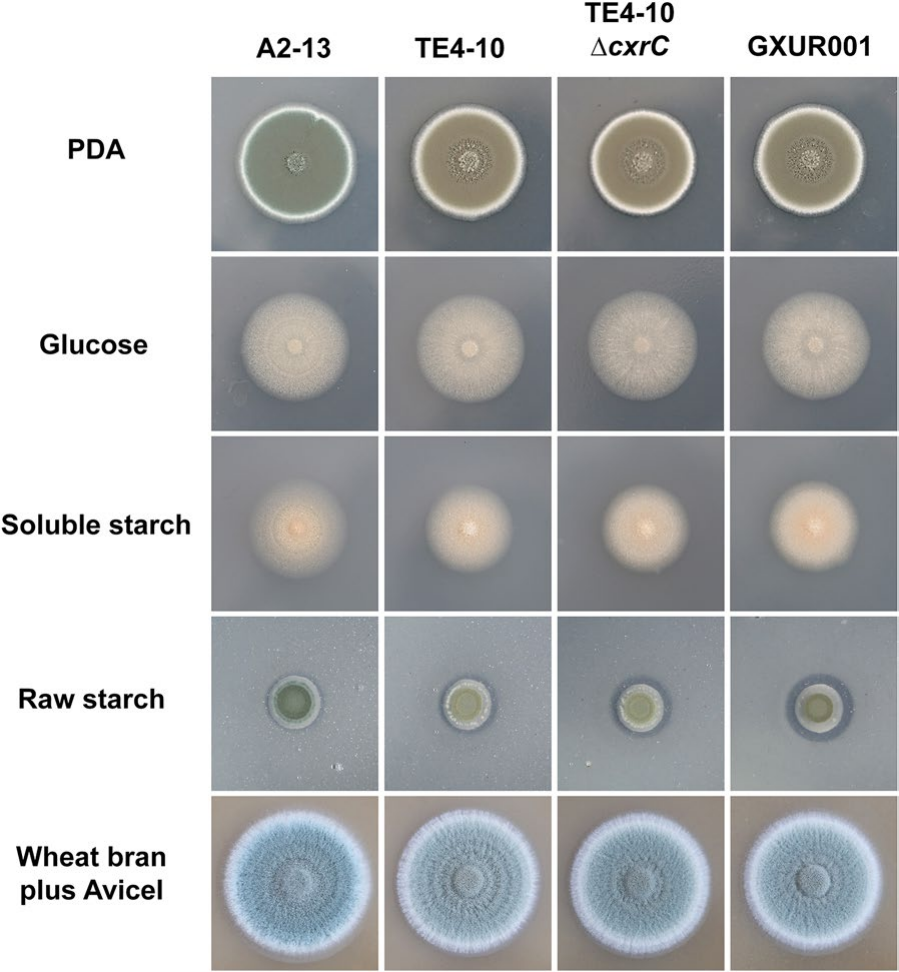
Phenotypic analysis of *Penicillium oxalicum* strains. Fungal strains were cultured for 4 days. PDA, potato dextrose agar

Colony phenotypes of the four *P. oxalicum* strains, A2-13, TE4-10, TE4-10Δ*cxrC* and GXUR001, on solid plates grown on several carbon sources were comparatively analysed after culturing for 4 days. The results revealed no significant differences in colony diameter of the four strains on all tested plates. However, *P. oxalicum* mutant GXUR001 showed the largest hydrolysis zone on plates with raw cassava flour. Colonies of TE4-10, TE4-10Δ*cxrC* and GXUR001 were yellow-green in colour on potato dextrose agar (PDA) and plates containing raw cassava flour, whereas colonies of the parent strain A2-13 were cyan. Compared with A2-13, the colony colour of TE4-10, TE4-10Δ*cxrC* and GXUR001 was lighter on plates with wheat bran plus Avicel (Fig. 4).

### Saccharification of raw starch flour by crude RSDE from engineered strain GXUR001

**Fig. 5:**
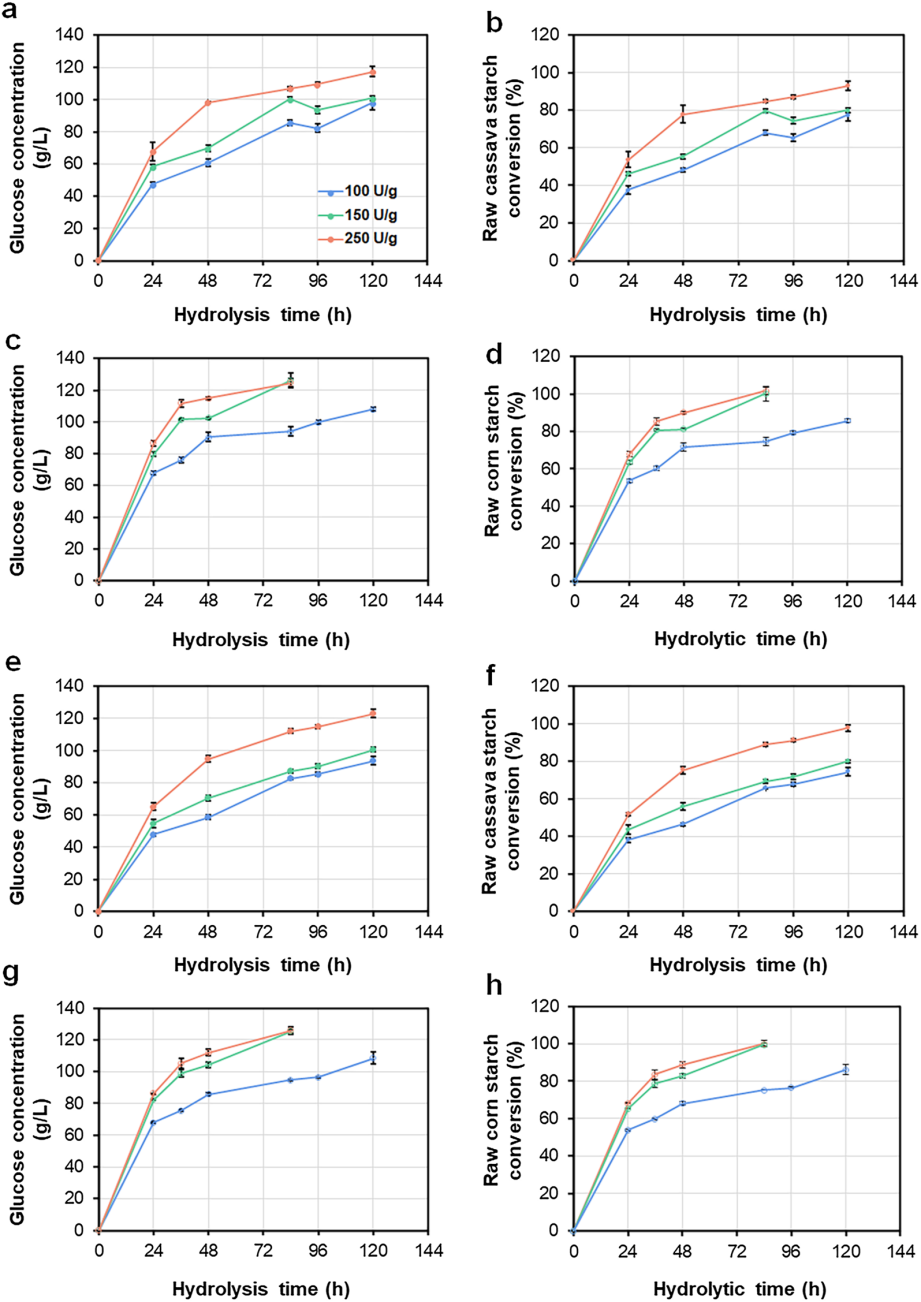
Saccharification of raw starch by engineered *Penicillium oxalicum* strain GXUR001 (**a**–**d**) and parent strain A2-13 (**e**–**h**). (**a**) Effect of GXUR001 RSDE loading on glucose released from hydrolysis of raw cassava flour. (**b**) Effect of GXUR001 RSDE loading on conversion of raw cassava flour. (**c**) Effect of GXUR001 RSDE loading on glucose released from hydrolysis of raw corn flour. (**d**) Effect of GXUR001 RSDE loading on conversion of raw corn flour. (**e**) Effect of A2-13 RSDE loading on glucose released from hydrolysis of raw cassava flour. (**f**) Effect of A2-13 RSDE loading on conversion of raw cassava flour. (**g**) Effect of A2-13 RSDE loading on glucose released from hydrolysis of raw corn flour. (**h**) Effect of A2-13 RSDE loading on conversion of raw corn flour. Crude RSDE were collected from fungal strains after 8 days culture on wheat bran plus Avicel. Saccharification was performed with 150 g/L of raw starch flour at 40 °C. Results are mean ± standard deviation, and each experiment is performed in three biological replicates

Currently, more than 50% of global bioethanol is made from corn starch as feedstock [1, 23]. Saccharification of raw starch is the key step during fermentation. Therefore, the saccharification efficiency of crude RSDE produced by GXUR001 was evaluated using raw cassava flour or raw corn flour. In this study, total starch contents contained in the raw cassava flour and raw corn flour were 75.6–78.7%, respectively. The results showed that the released glucose concentration following hydrolysis of raw cassava flour reached 117.2 g/L, with a starch conversion of 93.04% at 120 h, when carried out with 150 g/L loading of raw cassava flour at 40 °C and 250 U/g substrate (Fig. 5a, b). By contrast, under the same hydrolysis conditions, the released glucose concentration from raw corn starch reached 126.1 g/L with a starch conversion of 100% at 84 h at 150 U/g substrate (Fig. 5c, d). The hydrolysis ability of GXUR001 was comparable to that of the parental strain A2-13 (Fig. 5e–h).

Starch hydrolysis requires the appropriate and coordinated action of glucoamylase and α-amylase; α-amylase cleaves internal α-1,4-glycosidic linkages of starch chains, while glucoamylase breaks down α-1,4- and 1,6-glucosidic bonds at nonreducing ends to produce glucose [3, 4]. Raw starch granules are recalcitrant to amylase hydrolysis due to α-glucan packing and crystal allomorphs that are dependent on botanical origin. Interestingly, RSDE can digest raw starch granules to release glucose below the gelatinisation temperature via binding of starch granules with starch-binding domain. In general, cereal starch such as corn starch is susceptible, whereas unprocessed root starch such as cassava starch is resistant [20], and our results were consistent with this presumption. Additionally, the hydrolysis efficiencies of raw starch by crude RSDE from engineered strain GXUR001 and parental strain A2-13 showed no significant difference, suggesting that coordinated action among different kinds of amylases was similar.

## Conclusion

This study sequentially employed random mutagenesis and genetic engineering to enhance production of RSDE by *P. oxalicum*. The resulting GXUR001 strain achieved RSDE production of 252.6 U/mL using raw cassava flour, an increase of 42.4% relative to the parent strain A2-13. Both random mutagenesis and genetic engineering markedly upregulated the transcription of key amylase genes, including RSDG gene *PoxGA15A*. Moreover, crude RSDE from GXUR001 efficiently hydrolysed raw cassava flour and raw corn flour into glucose, with conversion values of 93.0% and 100%, respectively, comparable to those of A2-13. This mutant strain provides a potential source of RSDE for starch biorefining to produce bioethanol.

## Material and methods

### Fungal strains used in this study and culture conditions

*P. oxalicum* strains including the A2-13 parent strain [7] were grown on solid PDA for 5 days at 28 °C, and used for short-term preservation at 4 °C or reproduction.

To prepare crude RSDE, minimal modified medium containing carbon sources wheat bran (2%, w/v) and Avicel (3%, w/v) [7] was used to culture *P. oxalicum* strains for 8 days at 28 °C. Cultures were centrifuged for 10 min at 16,000 × *g* and 4 °C, and the obtained supernatant served as crude RSDE.

To extract total RNA for RT-qPCR assays, equal numbers of asexual spores from each *P. oxalicum* strain were inoculated into minimal modified medium containing glucose and cultured for 24 h. Hyphae were transferred into minimal modified medium containing wheat bran plus Avicel and culture was continued for 4–48 h.

### Plant materials and their pretreatments

Raw starch flours as hydrolysis substrates were purchased from a local farmers' market in Nanning, China, and processing was performed in accordance with a previous study [7].

### Mutagenesis

EMS mutagenesis was performed as reported previously [7]. Co^60^-γ-ray treatment of *P. oxalicum* spores was carried out by Guangxi Nanxiang Environmental Protection Co., Ltd. (Nanning, China) at doses of 0.4–3.0 KGy.

### Construction and verification of engineered *P. oxalicum* strains

*P. oxalicum* strains were genetically engineered based on homologous recombination [24] and confirmed via PCR using specific primers (Additional file 5: Table S2).

### Determination of RSDE activity

Measurement of RSDE activity was in accordance with a previously published procedure [8]. One RSDE activity unit (U) was the amount of enzyme required to release 1 μmol of reducing sugars per minute when hydrolysing raw cassava flour under specific conditions (pH 4.5 and 65 °C).

### RNA and DNA extraction

Total RNA from *P. oxalicum* was extracted using a DP419 RNA Extraction Kit (Tiangen Biochemical Technology Co., Ltd., Beijing, China) according to the manufacturer’s instructions. The DNA extraction referred to the method previously published [25].

### RNA-sequencing

RNA samples from *P. oxalicum* strains were submitted to Fraser Gene Information Co., Ltd. (Wuhan, China) for sequencing and further analysis according to previously described procedures [26]. Three biological replicates were included for each *P. oxalicum* strain.

### RT-qPCR

RT-qPCR was implemented based on a previous study [13], and the *POX_c04656* gene encoding actin served as an internal reference to calculate relative expression levels of each target gene. Expression levels in engineered *P. oxalicum* strains were normalised against levels in the parent strain. All experiments were repeated three times.

### Phenotypic survey

Spore suspensions of *P. oxalicum* strains were spread on solid plates supplemented with different carbon sources and cultured for 4 days at 28 °C. Carbon sources were raw cassava starch, soluble corn starch, wheat bran plus Avicel, PDA and glucose. Colony photographs were taken with an EOS-6D digital camera (Canon, Tokyo, Japan). A Cellsens Imaging system (Olympus, Tokyo, Japan) was also employed. All studies were performed in triplicate.

### Properties of crude RSDE

The methods for determining the optimum temperature and pH of crude RSDE, as well as the corresponding stability, were performed as previously reported [10].

### Hydrolysis of raw starch flours

Saccharification of raw cassava flour and raw corn flour was executed as previously described [10].

### Statistical analysis

Statistical analysis of the obtained data was conducted by one-way analysis of variance using SPSS (IBM, Armonk, NY, USA) and Student’s *t*-test using Microsoft Excel (Microsoft, Redmond, WA, USA).

### Data availability

Transcriptomic data was loaded into the Sequence Read Archive database, and the accession number GSE210161 was assigned.

## Supplementary Information

**Additional file 1: Figure S1.** Effects of physical–chemical mutagens on the growth of *Penicillium oxalicum* strains. (**a**) Lethality curve of mutant A2-13 treated by ethyl methanesulfonate. (**b**) Lethality curve of mutant TE2-23 treated by Co^60^-γ. (**c**) Lethality curve of mutant Co1-17 treated by Co^60^-γ. The final concentration of EMS used was 1.2%, and the concentration of spores loaded was 1 × 10^8^/mL.

**Additional file 2: Figure S2.** Schematic illustration (**a** and **b**) and PCR confirmation (**c** and **d**) of *Penicillium oxalicum* strains TE4-10∆*cxrC* and GXUR001. In panel c, the top panel shows PCR expression analysis of gene *PoxCxrC*; the middle and bottom panels show PCR verification of left-hand fragment and right-hand fragments. In panel d, the top part shows PCR expression analysis of genes *PoxCxrC-PoxAmyR*; the middle and bottom parts show PCR verification of the left-hand fragment and right-hand fragments. M, 1 kb DNA ladder; 1, ddH_2_O; 2, TE4-10; 3–5, transformants.

**Additional file 3: Figure S3.** Pearson’s correlations between transcriptomes from engineered strain GXUR001 and parental strain TE4-10. Fungal strains were cultured in medium containing wheat bran plus Avicel for 24 h after transfer from glucose.

**Additional file 4: Table S1.** Differentially expressed genes in engineered *Penicillium oxalicum* strain GXUR001 relative to TE4-10 cultured on wheat bran plus Avicel as carbon source.

**Additional file 5: Table S2.** Primers used in this study.

## Data Availability

All data generated or analysed during this study are included in this published article.
