# Supplementary figures and images for: Combination of genetic engineering and random mutagenesis for improving production of raw-starch-degrading enzymes in Penicillium oxalicum
Source: Microb Cell Fact. 2022 Dec 24;21:272. doi: 10.1186/s12934-022-01997-w (PMC9790131; doi:10.1186/s12934-022-01997-w)

**(a)**

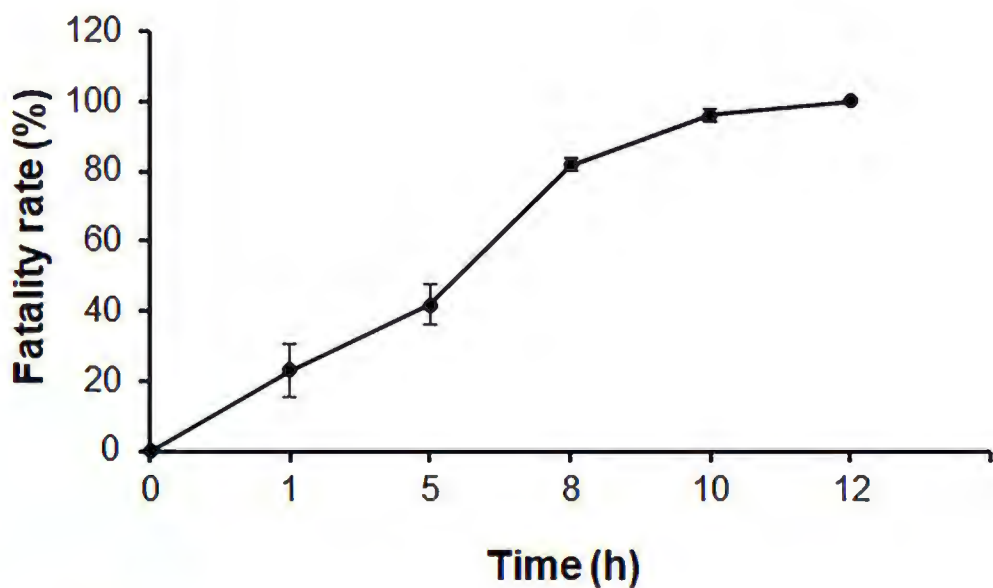

**(b)**

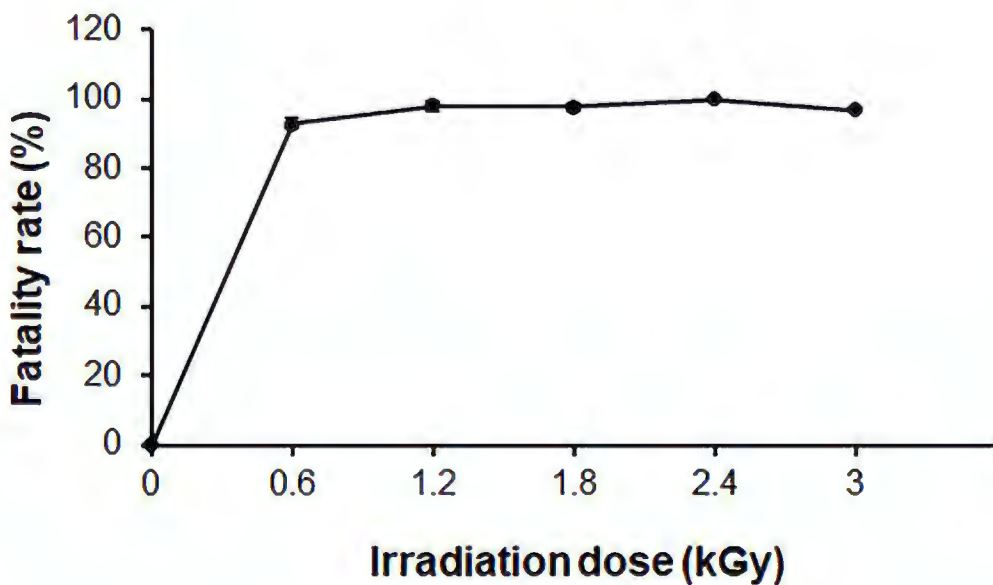

**(c)**

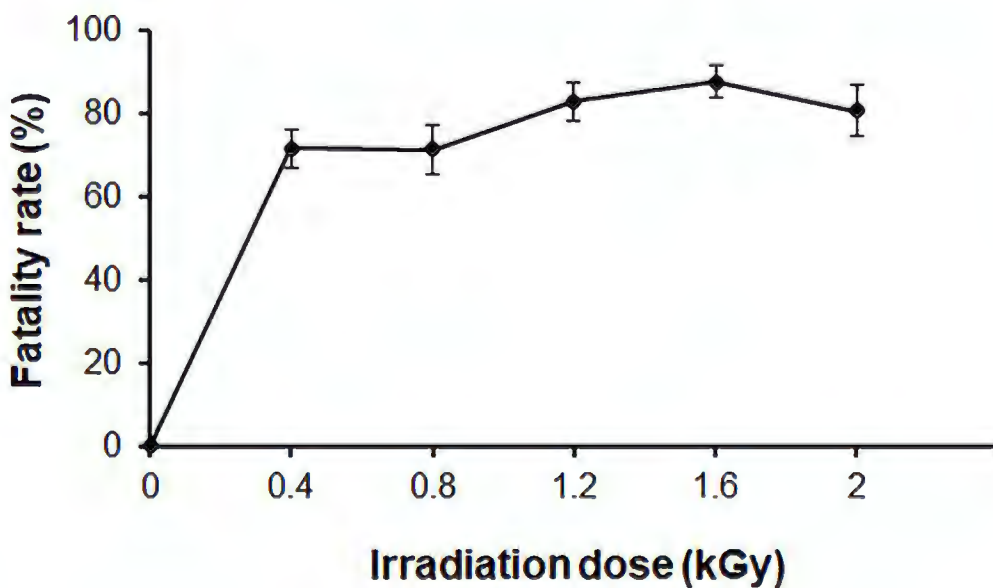

Supplement: Supplementary file 1 — Additional file 1: Figure S1. Effects of physical–chemical mutagens on the growth of Penicillium oxalicum strains. (a) Lethality curve of mutant A2-13 treated by ethyl methanesulfonate. (b) Lethality curve of mutant TE2-23 treated by Co60-γ. (c) Lethality curve of mutant Co1-17 treated by Co60-γ. The final concentration of EMS used was 1.2%, and the concentration of spores loaded was 1 × 10^8^/mL. [file 12934_2022_1997_MOESM1_ESM.pdf]

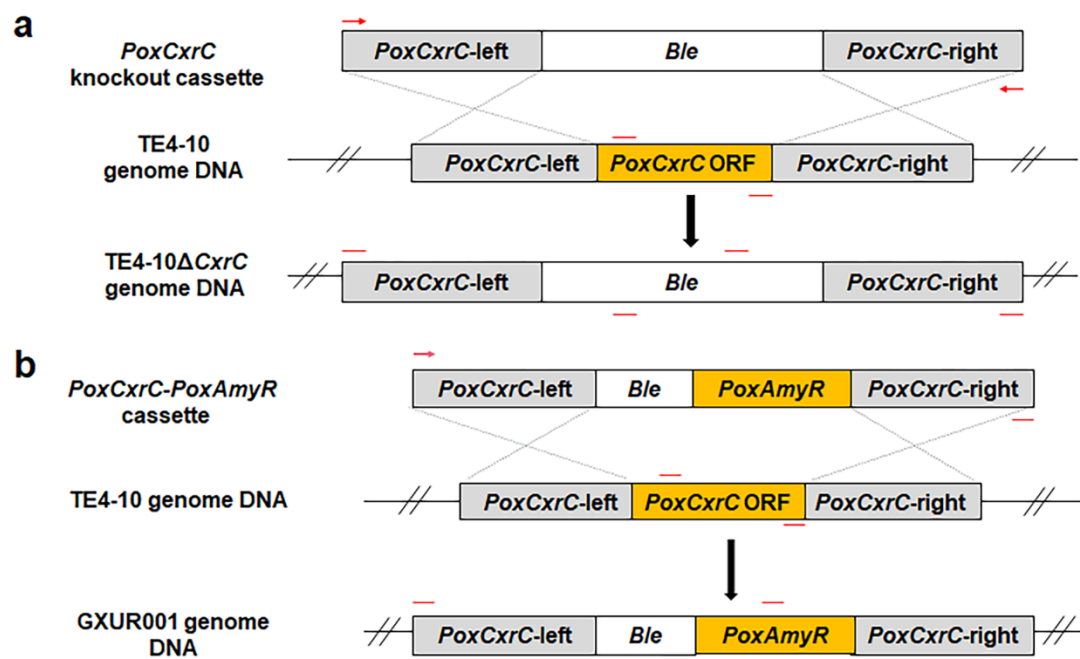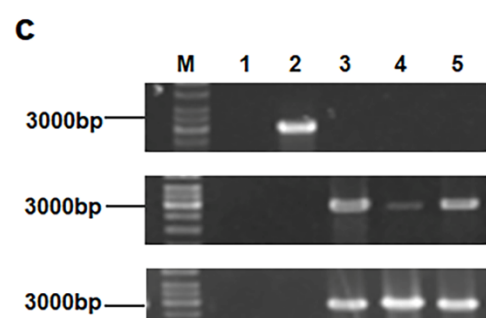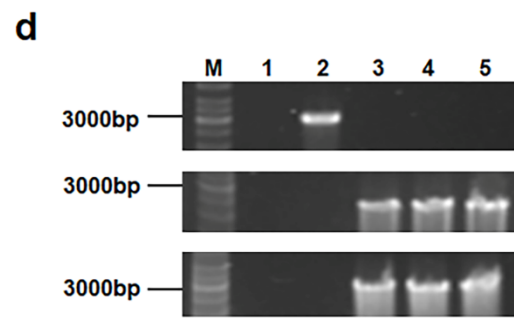

Supplement: Supplementary file 2 — Additional file 2: Figure S2. Schematic illustration (a and b) and PCR confirmation (c and d) of Penicillium oxalicum strains TE4-10∆cxrC and GXUR001. In panel c, the top panel shows PCR expression analysis of gene PoxCxrC; the middle and bottom panels show PCR verification of left-hand fragment and right-hand fragments. In panel d, the top part shows PCR expression analysis of genes PoxCxrC-PoxAmyR; the middle and bottom parts show PCR verification of the left-hand fragment and right-hand fragments. M, 1 kb DNA ladder; 1, ddH2O; 2, TE4-10; 3–5, transformants. [file 12934_2022_1997_MOESM2_ESM.pdf]
