# Supplementary material for: Combination of genetic engineering and random mutagenesis for improving production of raw-starch-degrading enzymes in Penicillium oxalicum
Source: Microb Cell Fact. 2022 Dec 24;21:272. doi: 10.1186/s12934-022-01997-w (PMC9790131; doi:10.1186/s12934-022-01997-w)
Supplement: Supplementary file 3 — Additional file 3: Figure S3. Pearson’s correlations between transcriptomes from engineered strain GXUR001 and parental strain TE4-10. Fungal strains were cultured in medium containing wheat bran plus Avicel for 24 h after transfer from glucose. [file 12934_2022_1997_MOESM3_ESM.pdf]

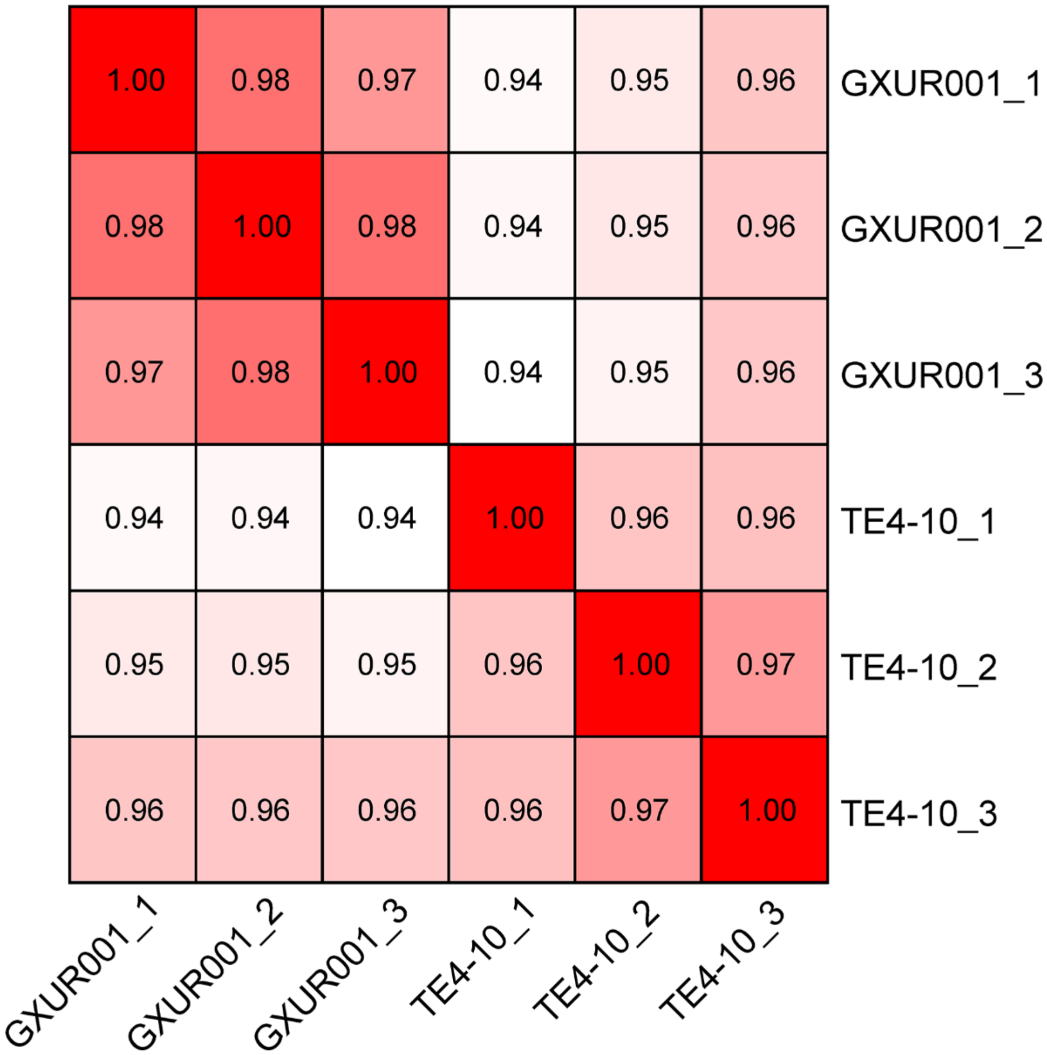
1.00 0.98 0.97 0.94 0.95 0.96 GXUR001_1
0.98 1.00 0.98 0.94 0.95 0.96 GXUR001_2
0.97 0.98 1.00 0.94 0.95 0.96 GXUR001_3
0.94 0.94 0.94 1.00 0.96 0.96 TE4-10_1
0.95 0.95 0.95 0.96 1.00 0.97 TE4-10_2
0.96 0.96 0.96 0.96 0.97 1.00 TE4-10_3
GXUR001_1
GXUR001_2
GXUR001_3
TE4-10_1
TE4-10_2
TE4-10_3
