# Supplementary material for: Combination of genetic engineering and random mutagenesis for improving production of raw-starch-degrading enzymes in Penicillium oxalicum
Source: Microb Cell Fact. 2022 Dec 24;21:272. doi: 10.1186/s12934-022-01997-w (PMC9790131; doi:10.1186/s12934-022-01997-w)
Supplement: Supplementary file 5 — Additional file 5: Table S2. Primers used in this study. [file 12934_2022_1997_MOESM5_ESM.pdf]

Additional file 5: Table S2 Primers used in this study

**Primer use**	**Primer name**	**Sequences (5'-3')**	**Size of PCR product (bp)**
**Primers used for the construction of *P. oxalicum* strain TE4-10Δ*cxrC***			
Construction of *CxrC* knokout cassette in TE4-10	PoxCxrC-LF	CTTGCTGCTTCCCCTGCCATA	2549
	PoxCxrC-LR	TTTAGAGGTAATCCTTCTTTCTAGAGGTCGGCGGTGTAAAGGAAATC	
	PoxCxrC-RF	TACCGCTGTTGAGATCCAGTGCCCCTTTGCCCCC	2775
	PoxCxrC-RR	TGACGGAGGTGAATCCAATAAG	
	PoxCxrC-NF	TTGGTTATGATTGGATTTGCC	5726
	PoxCxrC-NR	TCTCATAAACCAACGACGAAA	
	Ble-F	TCTAGAAAGAAGGATTACCTCTAAA	1733
	Ble-R	CTGGATCTCAACAGCGGTAA	
Verification of transformants	PoxCxrC-LF	CTTGCTGCTTCCCCTGCCATA	2947
	BlezuojiaoR	ACGAGATCAAGCAGATCAACG	
	PoxCxrC-MF	GCTATCATGCTGGGCCTTTT	2791
	PoxCxrC-MR	TCCGAGATCGGTCATTTGC	
	BleyuojiaoF	CTCTCAAGGATCTTACCGCT	2807
	PoxCxrC-RR	TGACGGAGGTGAATCCAATAAG	
**Primers used for the construction of *P. oxalicum* strain** GXUR001			

Construction of *CxrC*::*AmyR* overexpression cassette in TE4-10	PoxCxrC-LF	CTTGCTGCTTCCCCTGCCATA	2549
	PoxCxrC-LR	TTTAGAGGTAATCCTTCTTTCTAGAGGTCGGCGGTGTAAAGGAAATC	
	PoxCxrC-RF	TACCGCTGTTGAGATCCAGTGCCCCTTTGCCCCC	2775
	PoxCxrC-RR	TGACGGAGGTGAATCCAATAAG	
	PoxAmyR-NF	TACCGCTGTTGAGATCCAGGCGGTTGTGTAGTGTATTGTGG	3279
	PoxAmyR -NR	GATCCTACACATCGCCTCGAGATGGGCGAGTGGTGAAATAG	
	PoxCxrC-NF	TTGGTTATGATTGGATTTGCC	9005
	PoxCxrC-NR	TCTCATAAACCAACGACGAAA	
	Ble-F	TCTAGAAAGAAGGATTACCTCTAAA	1733
	Ble-R	CTGGATCTCAACAGCGGTAA	
Verification of transformants	PoxCxrC-LF	CTTGCTGCTTCCCCTGCCATA	2947
	BlezuojiaoR	ACGAGATCAAGCAGATCAACG	
	PoxCxrC-MF	GCTATCATGCTGGGCCTTTT	2791
	PoxCxrC-MR	TCCGAGATCGGTCATTTGC	
	PoxAmyRyoujiaoF	CACGGCATCGGAATGGTAAG	3344
	PoxCxrC-RR	TGACGGAGGTGAATCCAATAAG	
**Primers used for RT-qPCR**			
	RT-actin-F	ATGAGGCACAGTCTAAGCG	303
	RT-actin-R	CCAGAGTCCAGCACGATA	

	RT-amy13A-F	CTGACGGCTGCCCAATG	125
	RT-amy13A -R	CCAAATCGCAGTAAATCCC	
	RT-PoGA15A-F	CCTCGGTGAGCCCAAGTT	165
	RT-PoGA15A -R	CCAAAGTCAATCAAGGCAA	
	RT-amyR-F	ACCCAGCCAGGGAACCAC	132
	RT-amyR -R	CATTCCGATGCCGTGAGC	
	RT-Pox02412-F	TATGTGGATTCCTTCCGCTCTA	105
	RT-Pox02412-R	ATGGATTGCCTCCTTGGT	
